# Supplementary material for: Importance of occupation in the increase of gender differences in the advance of telework in Spain
Source: PLoS One. 2025 May 7;20(5):e0322847. doi: 10.1371/journal.pone.0322847 (PMC12058165; doi:10.1371/journal.pone.0322847)
Supplement: Attachment — Submitted filename: REVISION TABLES.pdf [file pone.0322847.s001.pdf]

```
. tab gender teletrabajo [aw= weight_core], row nof
Q2new [sex] Would you |
 describe yourself as |
   (transformed into |      teletrabajo
  gender_recoded for  |         0          1 |     Total
----------------------+----------------------+----------
                 Male |     82.96      17.04 |    100.00
               Female |     80.80      19.20 |    100.00
Or would you describe |     82.69      17.31 |    100.00
----------------------+----------------------+----------
                Total |     81.96      18.04 |    100.00

. tab gender teletrabajo if part_time==2 [aw= weight_core], row nof
Q2new [sex] Would you |
   (transformed into |      teletrabajo
  gender_recoded for  |         0          1 |     Total
----------------------+----------------------+----------
                 Male |     82.52      17.48 |    100.00
               Female |     77.42      22.58 |    100.00
Or would you describe |     77.02      22.98 |    100.00
----------------------+----------------------+----------
                Total |     80.46      19.54 |    100.00


. tab gender part_time, row nof
Q2new [sex] Would you |
 describe yourself as |  Q2d [part_time] And do you work part time
   (transformed into |               or full time?
  gender_recoded for  | Refusal (  DK (spont  Part time  Full time |     Total
----------------------+--------------------------------------------+----------
                 Male |      0.00       1.18       8.55      90.27 |    100.00
               Female |      0.07       0.66      23.53      75.75 |    100.00
Or would you describe |      0.00       0.00      22.22      77.78 |    100.00
----------------------+--------------------------------------------+----------
                Total |      0.03       0.93      15.67      83.36 |    100.00

. table hh_age_youngest_child gender [iw= weight_core], stat (mean
teletrabajo) nformat(%12.2fc)
-----------------------------------------
              |    Q2new [sex] Would you
              |     Male       Female
--------------+--------------------------
calculated    |
  age_12_15   |     0.15         0.22
  age_6_t0_11 |     0.17         0.23
  no children |     0.17         0.17
  younger_5   |     0.21         0.20
  Total       |     0.17         0.19
-----------------------------------------

. table hh_age_youngest_child gender [iw= weight_core], stat (mean parcial)
nformat(%12.2fc)
-----------------------------------------
              |    Q2new [sex] Would you
              |     Male       Female
--------------+--------------------------
calculated    |
  age_12_15   |     0.08         0.26
  age_6_t0_11 |     0.08         0.35
  no children |     0.10         0.23
  younger_5   |     0.09         0.42
  Total       |     0.10         0.28
-----------------------------------------
```
