# Supplementary material for: Importance of occupation in the increase of gender differences in the advance of telework in Spain
Source: PLoS One. 2025 May 7;20(5):e0322847. doi: 10.1371/journal.pone.0322847 (PMC12058165; doi:10.1371/journal.pone.0322847)
Supplement: Attachment — Submitted filename: Response to Reviewers.pdf [file pone.0322847.s002.pdf]

Response to editor & reviewers

Editor

Suggestion	Comment
The manuscript's analytical approach is too descriptive. It must explain in more detail the causal relationships that affect telework adoption, such as between teleworking and the proposed affecting conditions.	This can be explained for several reasons. Firstly, teleworking tends to be more conditioned by structural factors of employment, such as sector of activity, educational level and type of contract, than by personal or family situation. In other words, the possibility of teleworking depends more on the characteristics of the job than on the fact of living alone or with other people.<br><br>Secondly, the flexibility of teleworking allows both singles and people in couples or with dependents to adapt to this modality without their cohabitation structure representing a significant barrier. Unlike other work dynamics that may be more influenced by household composition, telework appears to be similarly accessible to different types of households. Likewise, these results could reflect a greater diversification of profiles within teleworking, in which different cohabitation groups manage to insert themselves without their personal situation clearly determining their participation in this type of work [line 257 and following]<br><br>This suggests that access to telework is not evenly distributed across all educational levels but is conditioned by educational background. It is possible that occupations that allow or facilitate this type of work require specialized skills, higher qualifications or belong to sectors where the use of digital technologies is essential. In this sense, telework could be reinforcing pre-existing inequalities in the labor market, favoring those with higher education and limiting access for those with lower educational levels [line 260 and following]<br><br>These results suggest that telework is not only linked to occupation, but also to job stability and workload. People with temporary contracts and reduced working hours, who tend to be in more precarious positions, have less access to this modality, which reinforces pre-existing labor inequalities. In addition, the fact that women have a greater presence in teleworking within these groups could be related to patterns of work-life balance and occupational segmentation, which raises questions about how teleworking may be reproducing gender dynamics in the labor market [line 271 and following]
There is a visible lack of figures and statistics related to the case of Spain. Therefore, a table showing the state of telework in Spain during the study's analysis period must be added.	The following amendments have been introduced (see line 167):<br>• A table showing Spain's teleworking data from 2012 to 2021 has been added<br>• Next text has been added: In the case of Spain (see Table 2), there has been a feminization of regular telework since 2020, as in the countries of the second pattern described above. Prior to this, the proportion of men and women teleworking was comparable, although in most years there were slightly more men than women teleworking.It was subsequent to the confinement due to the pandemic that the gap between men and women widened significantly. While the pre-pandemic period exhibited no significant disparity between men and women, the year 2020 marked an increase of 2.2 percentage points, and one percentage point by the year 2021. Therefore, the case of Spain exhibits a distinctive characteristic when compared to other countries within the same pattern. Consequently, the confinement and the pandemic resulted in a substantial increase in female teleworking, which was further fueled by the characteristics of the Spanish labor market, and gender variable has no major impact on the prevalence of telework (Duarte and Quirós, 2024; Eurofound, 2022a).
The proposed hypotheses must be formulated more explicitly to control for the proposed influential variables.	The following amendment has been introduced:<br>Text A has been replaced by Text B (see line 185):<br>Text A:<br>H.1.: Household composition acts as a moderator affecting the relationship between gender and propensity to telework, increasing in the case of women (Campdesuñer et al., 2023; Gohoungodji, 2022; Goldin, 2022; Ishino et al., 2022; Lyttelton et al., 2020; Pigini and Staffolani, 2019; Sun et al., 2023).<br><br>Text B:<br>H.1.: The composition of the household acts as a moderator in the relationship between gender and the propensity to telework, this influence being differentiated according to the presence or absence of children, as well as the form of cohabitation, whether living alone, with a partner or with more people. In households with children, the responsibilities of

	care and housework tend to fall on women, which increases their propensity to telework as a strategy to reconcile these demands (Campdesuñer et al., 2023; Lyttelton et al., 2020). On the other hand, in households without children, gender differences in the propensity to telework tend to be less pronounced, whereas in households with a couple or more people, assigned roles may amplify or reduce these differences (Goldin, 2022; Gohoungodji, 2023; Pigini and Staffolani, 2019; Ishino et al., 2021; Sun et al., 2023).<br><br>Text C has been replaced by Text D (see line 189):<br>Text C:<br>H.2.: Occupation acts as a moderating factor for gender differences in the propensity to telework (Asmussen et al., 2024; Chung and van der Lippe, 2020; Gálvez et al., 2021; Kley et al., 2023; López and Rodríguez, 2021; Lyttelton and Zang, 2020; Sostero et al., 2020; Lott et al., 2020).<br><br>Text D:<br>H.2.: Occupational category, defined by level of responsibility and skill, acts as a moderating factor in gender differences in the propensity to telework. Women in high-skill and higher-responsibility occupations are more likely to access telework due to the flexibility these positions often offer and the perception of trust involved in these roles (Kley y Reimer, 2023; Minkus et al., 2022; Chung and van der Lippe, 2020; Sostero et al., 2020). In contrast, in low-skilled occupations or occupations with lower levels of autonomy, gender differences in the propensity to telework are more marked, as these positions usually offer fewer options for work flexibility (Gálvez et al., 2021; López and Rodríguez, 2021).<br><br>Text E has been replaced by Text F (see line 193):<br>Text E:<br>H.3.: Education acts as a moderating factor in the propensity to telework (Kley and Reimer, 2023; Pigini and Staffolani, 2019; Santiago and Mergener, 2022).<br><br>Text F:<br>H.3.: Educational level acts as a moderating factor of the propensity to telework, differentiating access opportunities according to the degree of academic training. People with higher education are more likely to telework, given that the roles associated with this educational level tend to be less dependent on face-to-face work and more compatible with technological tools (Kley and Reimer, 2023; Pigini and Staffolani, 2019). In contrast, people with lower levels of education tend to occupy jobs that require manual skills or routine activities, limiting their ability to access teleworking
Although the logit model appears suitable for the paper's purposes, presenting marginal effects could offer a more intuitive result interpretation.	We have chosen to use a logistic regression model instead of presenting individual marginal effects for each variable, since our objective is to analyze the joint impact of the variables on the probability of teleworking. Marginal effects calculated in isolation may lead to misinterpretations, as they do not consider the interactions and relationships between the variables in the model.<br>To ensure a more accurate interpretation of the impact of each variable, we have presented the regression results in both tables and graphs, which allows us to visualize the actual effect of each predictor on the probability of teleworking, always controlling for the effect of the rest of the variables included in the model. This strategy provides a more rigorous and faithful view of the multivariate nature of the analyzed phenomenon.
Besides, additional diagnostic tests for logistic regression assumptions.	A logistic regression residual analysis was conducted to verify the validity of the logistic regression and the fulfillment of its assumptions. We have examined the behavior of the residuals using diagnostic plots, which show that the model is correctly specified and there are no systematic patterns suggesting fit problems.<br>The residual plots included in the analysis confirm that there are no indications of serious violations of the independence assumptions, absence of collinearity or poor model fit. In addition, the absence of influential outliers that could distort the estimates has been verified.<br>Thus, Graph 3 was included along with next text (see line 559):<br>Finally, an evaluation of the residuals of the logit model is conducted (Graph 3). It is observed that the residuals behave in accordance with the expected values, with no deviations identified, absence of collinearity or poor model fit.<br><br>Besides, in "Material and methods" section was included the next paragraph (see line 244):<br>Finally, an evaluation of the residuals of the logit model is conducted to verify the validity of the logistic regression and to determine whether the assumptions underlying the regression are fulfilled.

Discussion of measurement scales.	The measurement scales of different variables was pointed out (see lines 199, 208, 209, 216, 218, 224, 228 and 229)
The use of sample weights must be added.	The weights provided by the EWCTS survey are designed to ensure representativeness of the European population, correcting for possible biases derived from the sample design and non-response. However, given that our analysis focuses exclusively on Spain, the application of these weights may not be appropriate. The sampling structure and nonresponse patterns may differ within the Spanish subgroup, which would make the weights designed at the European level not correctly reflect the distribution of the Spanish population.<br>Furthermore, the use of these weights in a smaller sample such as ours carries a significant risk of inflating the type I error rate (false positives) by artificially reducing variances and making virtually all variables appear statistically significant. This is because the weights amplify the contribution of certain observations, affecting the estimation of standard errors in regression models.<br>According to methodological references, in studies whose main objective is to analyze relationships between variables, rather than to produce descriptive population estimates, the inclusion of weights is not always necessary and can even be detrimental if the weights have not been designed specifically for the subgroup analyzed. A valid alternative is to correctly specify the model by including the variables that affect the probability of response, which we have done by incorporating factors such as age, sex, region, occupation and sector in the analysis, as recommended by the EWCTS itself.
The reduction in the sample size from 2,903 to 1,320 individuals must be adequately explained.	The reduction of the sample size from 2,903 to 1,320 individuals is due to a refinement process based on consistency with the objectives of the study. In the original EWCTS survey, responses are distributed across multiple levels, and in some cases, the categorization is not clear or does not directly fit the variables in our model.<br>To avoid arbitrary subjective decisions and not artificially inflate the sample size, we have chosen to exclude those cases in which the responses could not be precisely assigned to the categories established in our analysis. This procedure guarantees the validity of the results and consistency in the classification of the variables, avoiding possible biases derived from an ambiguous assignment of responses.<br><br>The following amendment has been introduced, replacing text A by text B (see line 174):<br>Text A:<br>As a result of including the variables relevant to our analysis and selecting the data specific to Spain, we obtained a final sample size of 1,320 individuals.<br><br>Text B:<br>As a result of a refinement process based on consistency with the objectives of the study, the relevant variables and cases to our analysis were included, we obtained a final sample size of 1,320 individuals.
The empirical work needs to present results for the entire and gender-specific samples.	Following text has been introduced along with the new Table 4 regarding the entire sample analysis (see line 275):<br><br>The results of the logistic regression analysis for both men and women (Table 4) indicate the existence of high statistical significance of the education variable ("higher education level": p<.001; z=7.959), and the labor variable such as "indefinite" (p<.001; z=5.073) related to "contract type". Nevertheless, the other labor variable such as "full time" (p=.014; z=2.446) linked to "working time" has low statistical significance. The analysis also examined the association between other labor variable such as "occupation type" and telework, focusing on the occupation of scientific professionals, that have a high probability of telework for both men and women (0.91 ± 0.63 and 0.94 ± 0.74, respectively). All occupations under consideration have high Wald statistics and negative values, apart from "service and sales workers", which do not demonstrate statistical significance.<br>Conversely, the relational variables such as having "children" (p=.55; z=0.599) and "living with a partner and more people" (p=.213; z=1.246) lack statistical significance in relation to telework.<br>Consequently, education and labor variables have been demonstrated to exert a substantial influence on telework patterns, indicating that workers with high level of education and indefinite contracts exhibit a greater propensity to telework, while those with full-time contracts demonstrate a slightly lower likelihood of doing so. On the other hand, although the labor variable related to occupation has a significant contribution to the predictive model of telework, it does so with an inverse character. This implies that the different occupations considered show a low probability to telework considering the occupation of scientific professionals, as a reference. Finally, the relational variables do not contribute to the predictive model related to telework.<br><br>Next text has been introduced along with the new Table 6 regarding the entire sample analysis (see line 307):

	The table 6 presents the ratios and confidence intervals of the variables considered in the telework predictor model, considering both men and women samples. It indicates that the variables "education level-higher", "indefinite" related to the type of contract, and "full-time" referred to the type of working time have odds ratios greater than 1. This finding suggests that they directly influence the occurrence of teleworking. Besides, the strength of the relationship is more robust in the case of the "education level-higher" variable (OR=5.03) compared to the "indefinite" variable (OR=3.35) and the "full-time" variable (OR=1.76).<br>Conversely, the labor variables related to occupation have odds ratios of less than 1, so their influence on teleworking is inverse. Especially significant is the strength of the inverse relationship shown by the occupations "skilled agricultural, forestry and fishery workers", "plant and machine operators, and assemblers", "Elementary occupations", and "armed forces occupations" with telework. This indicates that the probability of teleworking in these occupations is very low.<br>Finally, the test of multicollinearity, based on the Generalized Variance Inflation Factors (GVIF) indicates that there are no collinearity problems as their value is close to 1.
Some results in Tables 3 and 4 show striking gender differences that require further explanation.	Regarding Table 3, the excerpt A has been replaced by the excerpt B (see line 299):<br>A:<br>In contrast to men, it is possible to add other variables with statistical significance such as the premises "women working full-time" ($p<.05$; z=2.092) and "technicians and associate professionals" ($p<.001$; z=-2.622).<br>B:<br>In contrast to men, it is possible to indicate some differences regarding labor variables. Firstly, the variable "indefinite" related to type of contract has less statistical significance for women sample ($p<.01$; z=2.761) than for men ($p<.001$; z=4.723). This indicates that having an indefinite contract and teleworking is more likely for men than for women. Secondly, the variable "full-time" related to working time exhibits statistical significance for women ($p<.05$; z=2.092), while it is not significant for men. These findings indicate that full-time employment and teleworking are more prevalent among women than among men. Finally, in this final set of occupation-related variables, both the variable "clerical support workers" and "elementary occupations" exhibit lower degrees of statistical significance in the case of women than in the case of men. Consequently, in these occupations, women exhibit a lower propensity to telework than men. Nevertheless, it is noteworthy that the variable "technicians and associate professionals" related to occupation is statistically significant only for women ($p<.01$; z=-2.622), suggesting that those engaged in such occupations are more prone to telework than their male counterparts.<br><br>Regarding Table 4, the excerpt A has been replaced by the excerpt B (see line 313):<br>A:<br>The same results can be observed among women, but, in their case, the predictor related to "full-time", has an odds ratio bigger than 1. Therefore, this variable should be added to the previously mentioned ones, whose influence on the propensity to telework is direct.<br>B:<br>The results for the sample of women are similar, since the variables of level of higher education and permanent contract have odds ratios greater than 1, while the labor variables referring to occupation have odds ratios lower than 1. Firstly, the assumption of full-time work has an odds ratio higher than 1 in the case of women, so it is a descriptor to be added in the explanation of female teleworking while it is not relevant in the case of men. Secondly, the variable "indefinite" related to the type of contract exhibits a stronger association with telework among men (OR=6.70) compared to women (OR=2.44). Thirdly, higher education level emerges as a more robust predictor for men (OR=5.75) compared to women (OR=4.85). Finally, in the context of the variables associated with occupation, it is noteworthy that the value of the odds ratio for the occupation "service and sales workers" is 0.35 for men, while for women it approaches 1 (OR=0.90), indicating that its effect on female teleworking is virtually negligible.
The study's timing during the pandemic should be emphasized by specifying when the effects were recorded.	The following amendment has been introduced (see line 172):<br>The fieldwork was conducted from March to November of 2021, thus marking one year after the initial onset of the confinement measures implemented in response to the pandemic. Consequently, the repercussions of the pandemic on telework practices were recorded in this edition of the EWCS.
The construction of the dichotomous variable for teleworking must be precisely explained.	According to Eurofound and EWCTS 2021, based on the question "How often have you worked in any of the following locations during the last 12 months?" with five response options, the variable "telework" was constructed by Eurofound, based on three variables:

	• ICT should be used either 'always' or 'often';<br>• employees' job must be teleworkable;<br>• and employees should have been working from home either 'rarely', 'sometimes', 'often' or 'always'.<br>This variable was then recoded for cases where these conditions were met with the following categories (all of them were used by the authors):<br>• Full-time telework,<br>• Partial telework,<br>• Occasional telework,<br>• Respondents working only from the employer's premises.<br><br>Regarding the use of the dummy variable from the original variable that asks: "How often have you worked in the following places? YOUR OWN HOME (Never, Rarely, Sometimes, Often, Always), we did not want to assume that telework always takes place at the workers' home, but rather that it was all telework that can take place outside the employer's premises or on the employer's premises, although with a significant degree of teleworkability.<br>Consequently, given that the dummy variable developed by Eurofound encompassed this consideration, we determined that its implementation would be optimal.<br><br>The excerpt A has been replaced by the excerpt B (see line 199 and following):<br>A:<br>Based on this question, we have considered the variable that Eurofound has constructed with the following values: Full-time telework (employees always work from home); Partial telework (employees often work from home and employees often working from home and from their employer's premises at least sometimes); Occasional telework (sometimes or rarely work from home), Employer's premises with some degree of teleworkability (employees always working from their employer's premises in jobs with some degree of teleworkability).<br><br>B:<br>Based on this question with five responses options (never, rarely, sometimes, often and always), the telework variable considered as a nominal and constructed by Eurofound was based on three variables (ICT should be used either 'always' or 'often'; employees' job must be teleworkable; and employees should have been working from home either 'rarely', 'sometimes', 'often' or 'always'). The resulting variable was then recoded with the following values: Full-time telework (employees always work from home); Partial telework (employees often work from home and employees often working from home and from their employer's premises at least sometimes); Occasional telework (sometimes or rarely work from home), Employer's premises with some degree of teleworkability (employees always working from their employer's premises in jobs with some degree of teleworkability).
The general presentation must be improved. Revise manuscript sections, complete tables with standard logistic regression statistics, and include titles and sources for graphs.	The presentation has been improved by numbering manuscript sections, completing tables with standard logistic regression statistics and sample sizes, and including titles and sources for the two final graphs.<br><br>The following amendment has been introduced in Table 1 (see line 164):<br>Note: The values correspond to the subtraction of the percentage of men and women who telework. A positive value indicates that the proportion of men is higher than women one, while a negative value indicates that the proportion of women is higher than men one.
The paper's limitations must be mentioned by adding comments to acknowledge the study's constraints.	This research opens interesting avenues of work the at allow further deepening the understanding of telework as a social and labor phenomenon. In particular, it would be relevant to explore in more detail the unequal development of teleworking from a gender perspective, considering the differences that may arise depending on the type of work dedication, whether full or part-time. Future research could delve deeper into how work dynamics in telework environments affect men and women differently, revealing possible structural inequalities and specific patterns that have not been addressed in depth in this paper.<br>Another valuable line of research would be to detail how these gender differences in telework relate to family composition, particularly in households with children. In this sense, it could be of particular interest to study the impact of telework as a function of the age of the children, since care needs and family organization vary significantly according to this variable. This approach would make it possible to identify more precisely how teleworking influences family and work responsibilities,

	and how these interactions may be a key factor in perpetuating or reducing gender inequalities in the work and domestic spheres. However, these questions are beyond the scope of this paper and are proposed as future lines of research.
The conclusion section must be refined by separating and strengthening it, ensuring it reflects the refined analysis and addresses policy implications based on the findings.	The Discussion and Conclusions sections have been separated.
Finally, the manuscript must homogenize the use of American English in the text, such as substituting the word "labor" instead of "labour" and using decimal points instead of commas to describe numerical data.	The following amendment has been introduced:<br>• "labour" has been changed to "labor" in following lines: 15, 21, 38, 41, 47, 58, 59, 69, 95, 117, 127, 164, 273, 400, 410, 414, 417, 454, 455, 487, and on Figure 1.<br>• "relativise" has been changed to "relativize" (line 39)<br>• "generalise" has been changed to "generalize" (line 322)<br>• "flexibilisation" has been changed to "flexibilization" in following lines: 127, 415, 416<br>• Decimal points have been changed on Tables 1 & 2, and in lines 172 & 174
The study could have provided a comparative analysis with other EU countries to get additional insights into the uniqueness of the Spanish case.	The Spanish experience regarding telework after the pandemic has been especially distinctive compared to other European Union countries, which makes this country a singular case worthy of analysis.<br><br>The following amendments have been introduced in lines 156-157, to highlight the distinctive case of Spain:<br>• "…, including Ireland, Sweden, Finland, the Netherlands, Italy, Lithuania, and even Spain, with some distinctive features (see Table 2)."<br>• Delete the sentence: "A similar evolution has been found in Spain although its gender gap was minimal until 2020."
Besides, investigating the quality of telework experiences across different occupations and genders would have also provided valuable insights	Included in limitations and future lines (See line 496 and following)
Although the manuscript proposes three axes of analysis to explain the telework phenomenon in Spain (i.e., productive strategy, labor relations, and psychosocial consequences), there is a very superficial academic analysis of teleworking and its evolution in the wake of the COVID-19 pandemic.	Following text has been added (see line 168):<br>In this context, the discussion about the possibilities of telework during the Covid-19 pandemic intensified. As highlighted above, there was a shift from limited to intensive use in order to maintain economic activity (Belzunegui-Eraso and Erro, 2020; Rimbau, 2021). At the time of the telework boom in Spain, the perception of Spanish workers (CIS, 2021) was that telework was inevitable due to its unstoppable trend, since although only one third of the Spanish workforce would be able to telework due to the nature of their job (Anghel *et al*., 2020), 28.8% thought that the number of jobs that would be performed by means of telework would increase.<br>With slight changes, the profile of teleworkers in the European Union and Spain during the Covid-19 pandemic was maintained with respect to the previous period (Rodríguez-Pasquín *et al*., 2024). This was a highly educated or white-collar worker, working full-time with a permanent contract, mainly in the service sector (Milasi *et al*., 2020; Sostero *et al*., 2020). On the other hand, a low level of innovation has been observed in the organization of telework, maintaining in the virtual environment practices typical of the analog environment (Molina et al., 2021), although it has shown an important use of labor flexibilization after the approval of Law 10/2021 on telework (Duarte and Quirós, 2024).
Another point concerns the comment that COVID-19 has altered the digitalization process of modern societies; however, there is a visible lack of discussion, comments and criticisms on how the pandemic has changed that process.	Following text has been added (see line 33):<br>The COVID-19 pandemic has triggered current digital shift (Manzanera-Román and Brändle, 2022), which has specific characteristics compared to earlier digital shifts, as speed, intensity and high capacity to penetrate daily habits and practices, reaching a substantial transformation of previous social structures and social forms (Cámara *et al*., 2017; Torres, 2017). The pandemic had a notable effect on the acceleration and expansion of this phenomenon, making digital technologies essential for maintaining daily life in lockdown, especially (Fundación BBVA, 2021). The perception of the great importance of digital technologies is projected into the future, marking a turning point, as 56.4% of Spaniards believed that they will play an even more important role in their lives once the health crisis was over and normality is restored (Centro de Investigaciones Sociológicas, 2021). In European Union, up to 81.0% considered that digital technologies would be important in their lives by 2030.

Reviewer #1

Suggestion	Comment
1. Research Hypotheses<br>The article analyzes the factors influencing the likelihood of teleworking in Spain. The three research hypotheses proposed are somewhat simplistic. While they allow for a descriptive examination of how household composition, occupation, and education affect the probability of teleworking, it would be advisable to go further and formulate more sophisticated hypotheses. These could consider the necessity of controlling for household composition, educational attainment, and occupation—variables that common sense suggests are influential in teleworking.	H.1.: The composition of the household acts as a moderator in the relationship between gender and the propensity to telework, this influence being differentiated according to the presence or absence of children, as well as the form of cohabitation, whether living alone, with a partner or with more people. In households with children, the responsibilities of care and housework tend to fall on women, which increases their propensity to telework as a strategy to reconcile these demands (Campdesuñer et al., 2023; Lyttelton et al., 2020). On the other hand, in households without children, gender differences in the propensity to telework tend to be less pronounced, whereas in households with a couple or more people, assigned roles may amplify or reduce these differences (Goldin, 2022; Gohoungodji, 2023; Pigini and Staffolani, 2019; Ishino et al., 2021; Sun et al., 2023).<br><br>H.2. Occupational category, defined by level of responsibility and skill, acts as a moderating factor in gender differences in the propensity to telework. Women in high-skill and higher-responsibility occupations are more likely to access telework due to the flexibility these positions often offer and the perception of trust involved in these roles (Chung and van der Lippe, 2020; Sostero et al., 2020). In contrast, in low-skilled occupations or occupations with lower levels of autonomy, gender differences in the propensity to telework are more marked, as these positions usually offer fewer options for work flexibility (Gálvez et al., 2021; López and Rodríguez, 2021). Moreover, in roles of medium and high responsibility, telework may be perceived as a mechanism to manage work and personal demands, especially benefiting women who assume greater domestic responsibilities (Lyttelton and Zang, 2020; Kley et al., 2023). However, in lower-skilled occupations, where tasks often require face-to-face attendance, telework tends to be less accessible, reinforcing gender barriers in the workplace.<br><br>H.3. Educational level acts as a moderating factor of the propensity to telework, differentiating access opportunities according to the degree of academic training. People with higher education are more likely to telework, given that the roles associated with this educational level tend to be less dependent on face-to-face work and more compatible with technological tools (Kley et al., 2023; Pigini and Staffolani, 2019). In contrast, people with lower levels of education tend to occupy jobs that require manual skills or routine activities, limiting their ability to access teleworking [line 185 and following]
2. Methodology<br>The use of the logit model is suitable for describing the variables associated with teleworking. As a suggestion, presenting marginal effects could offer a more intuitive interpretation of the results.	We have chosen to use a logistic regression model instead of presenting individual marginal effects for each variable, since our objective is to analyze the joint impact of the variables on the probability of teleworking. Marginal effects calculated in isolation may lead to misinterpretations, as they do not consider the interactions and relationships between the variables in the model.<br>To ensure a more accurate interpretation of the impact of each variable, we have presented the regression results in both tables and graphs, which allows us to visualize the actual effect of each predictor on the probability of teleworking, always controlling for the effect of the rest of the variables included in the model. This strategy provides a more rigorous and faithful view of the multivariate nature of the analyzed phenomenon.
3. Descriptive Tone<br>The overall tone of the article is somewhat descriptive. Could the analysis have explored causal relationships, for example, between teleworking and job satisfaction (or stress, isolation, etc.)? Does the survey include data on these or other variables (e.g., burnout, well-being, work-life balance)? The literature review addresses many of these issues, which are not subsequently investigated in the empirical work. For instance, the review mentions potential increases in job satisfaction due to better time management or reductions in opportunities for social relationships. Later, it is stated that teleworking may deteriorate working conditions. Investigating satisfaction levels could offer insights into whether teleworking is associated with	This is an interesting variable present in the database (Q44) and was even considered in the initial stages of the research. However, we finally considered that it was not aligned with the final objectives of the research. These were oriented to the explanation of the propensity to telework by gender considering family factors versus work factors. Actually, we consider that this is a limitation that has been reflected in the corresponding section.

deteriorated job satisfaction, working conditions, or work-life balance.	
4. Selection Bias and Causal Effects<br>If the authors consider studying the causal effect of teleworking on satisfaction or work-life balance, it is recommended to control for selection bias. Men and women tend to work in different occupations, with men overrepresented in manual jobs and women in white-collar jobs.	Since it has been considered as a limitation and reflected appropriately, it will be considered in future research.
5. Sample Size Reduction<br>The drastic reduction in sample size from 2,903 observations in the original database to only 1,320 individuals in the final sample is not well-explained. The article refers to the inclusion of relevant variables for analysis and an (unjustified) selection of specific Spanish data.	The reduction of the sample size from 2,903 to 1,320 individuals is due to a refinement process based on consistency with the objectives of the study. In the original EWCTS survey, responses are distributed across multiple levels, and in some cases, the categorization is not clear or does not directly fit the variables in our model.<br>To avoid arbitrary subjective decisions and not artificially inflate the sample size, we have chosen to exclude those cases in which the responses could not be precisely assigned to the categories established in our analysis. This procedure guarantees the validity of the results and consistency in the classification of the variables, avoiding possible biases derived from an ambiguous assignment of responses.<br><br>The following amendment has been introduced, replacing text A by text B (see line 174):<br>Text A:<br>As a result of including the variables relevant to our analysis and selecting the data specific to Spain, we obtained a final sample size of 1,320 individuals.<br><br>Text B:<br>As a result of a refinement process based on consistency with the objectives of the study, the relevant variables and cases to our analysis were included, we obtained a final sample size of 1,320 individuals.
6. Gender-Specific Estimates<br>The empirical work could have presented estimation results for the entire sample (men and women) in addition to the two gender-specific samples, including a dummy variable to capture the effect of being female on the likelihood of teleworking.	Next text has been introduced along with the new Table 4 regarding the entire sample analysis (see line 275):<br><br>The results of the logistic regression analysis for both men and women (Table 4) indicate the existence of high statistical significance of the education variable (“higher education level”: p<.001; z=7.959), and the labor variable such as “indefinite” (p<.001; z=5.073) related to “contract type”. Nevertheless, the other labor variable such as “full time” (p=.014; z=2.446) linked to “working time” has low statistical significance. The analysis also examined the association between other labor variable such as “occupation type” and telework, focusing on the occupation of scientific professionals, that have a high probability of telework for both men and women (0.91 ± 0.63 and 0.94 ± 0.74, respectively). All occupations under consideration have high Wald statistics and negative values, apart from “service and sales workers”, which do not demonstrate statistical significance.<br>Conversely, the relational variables such as having “children” (p=.55; z=0.599) and “living with a partner and more people” (p=.213; z=1.246) lack statistical significance in relation to telework.<br>Consequently, education and labor variables have been demonstrated to exert a substantial influence on telework patterns, indicating that workers with high level of education and indefinite contracts exhibit a greater propensity to telework, while those with full-time contracts demonstrate a slightly lower likelihood of doing so. On the other hand, although the labor variable related to occupation has a significant contribution to the predictive model of telework, it does so with an inverse character. This implies that the different occupations considered show a low probability to telework considering the occupation of scientific professionals, as a reference. Finally, the relational variables do not contribute to the predictive model related to telework.<br><br>Next text has been introduced along with the new Table 6 regarding the entire sample analysis (see line 307):<br>The table 6 presents the ratios and confidence intervals of the variables considered in the telework predictor model, considering both men and women samples. It indicates that the variables "education level-higher", "indefinite" related to the type of contract, and "full-time" referred to the type of working time have odds ratios greater than 1. This finding suggests that they directly influence the occurrence of teleworking. Besides, the strength of the relationship is more robust in the case of the "education level-higher" variable (OR=5.03) compared to the "indefinite" variable (OR=3.35) and the "full-time" variable (OR=1.76).

	Conversely, the labor variables related to occupation have odds ratios of less than 1, so their influence on teleworking is inverse. Especially significant is the strength of the inverse relationship shown by the occupations “skilled agricultural, forestry and fishery workers”, “plant and machine operators, and assemblers”, “Elementary occupations”, and “armed forces occupations” with telework. This indicates that the probability of teleworking in these occupations is very low.<br>Finally, the test of multicollinearity, based on the Generalized Variance Inflation Factors (GVIF) indicates that there are no collinearity problems as their value is close to 1.
7. COVID-19 Context<br>It should be emphasized that 2021 was still heavily influenced by the COVID-19 pandemic (the second major wave occurred in 2022).	The following amendment has been introduced (see line 172):<br>The fieldwork was conducted from March to November of 2021, thus marking one year after the initial onset of the confinement measures implemented in response to the pandemic. Consequently, the repercussions of the pandemic on telework practices were recorded in this edition of the EWCS.
8. Definition of Key Variables<br>The definition of the variable of interest is not precisely explained. While the article mentions the source variable, it does not detail how the dichotomous variable was constructed. It refers to working with a variable created by Eurofound (Full-time teleworking, Part-time teleworking, Occasional teleworking, and On-site work with some degree of teleworkability). Were all categories used or only some of them? It might be simpler to construct the dummy variable from the original variable that asks: "How often have you worked in the following places? YOUR OWN HOME (Never, Rarely, Sometimes, Often, Always)."	According to Eurofound and EWCTS 2021, based on the question “How often have you worked in any of the following locations during the last 12 months?” with five response options, the variable “telework” was constructed by Eurofound, based on three variables:<br>• ICT should be used either 'always' or 'often';<br>• employees' job must be teleworkable;<br>• and employees should have been working from home either 'rarely', 'sometimes', 'often' or 'always'.<br>This variable was then recoded for cases where these conditions were met with the following categories (all of them were used by the authors):<br>• Full-time telework,<br>• Partial telework,<br>• Occasional telework,<br>• Respondents working only from the employer's premises.<br><br>Regarding the use of the dummy variable from the original variable that asks: "How often have you worked in the following places? YOUR OWN HOME (Never, Rarely, Sometimes, Often, Always), we did not want to assume that telework always takes place at the workers' home, but rather that it was all telework that can take place outside the employer's premises or on the employer's premises, although with a significant degree of teleworkability. Consequently, given that the dummy variable developed by Eurofound encompassed this consideration, we determined that its implementation would be optimal.<br><br>The excerpt A has been replaced by the excerpt B (see line 199 and following):<br>A:<br>Based on this question, we have considered the variable that Eurofound has constructed with the following values: Full-time telework (employees always work from home); Partial telework (employees often work from home and employees often working from home and from their employer’s premises at least sometimes); Occasional telework (sometimes or rarely work from home), Employer’s premises with some degree of teleworkability (employees always working from their employer’s premises in jobs with some degree of teleworkability).<br><br>B:<br>Based on this question with five responses options (never, rarely, sometimes, often and always), the telework variable constructed by Eurofound was based on three variables (ICT should be used either ‘always’ or ‘often‘; employees’ job must be teleworkable; and employees should have been working from home either ‘rarely’, ‘sometimes’, ‘often’ or ‘always’). The resulting variable was then recoded with the following values: Full-time telework (employees always work from home); Partial telework (employees often work from home and employees often working from home and from their employer’s premises at least sometimes); Occasional telework (sometimes or rarely work from home), Employer’s premises with some degree of teleworkability (employees always working from their employer’s premises in jobs with some degree of teleworkability).

9. Descriptive Tables<br>Some results from the descriptive tables are striking. For example, in Table 2, there are significant gender differences in teleworking among full-time employees (42.7% for men versus 62.3% for women), figures that are inconsistent with the descriptives in Table 1.<br>Similarly, lines 247 to 250 compare the proportion of teleworkers by gender and the presence of children in the household. The proportions shown are surprisingly high: the percentage of men teleworking is around 40%, rising to 50% for women. The drastic reduction in sample size might explain these unusual results (e.g., "regarding the "children" variable, the proportion of teleworkers with and without children is very similar for men (42.4% and 39.8%, respectively) and women (56.1% and 50.0%, respectively)").	This can be explained for several reasons. Firstly, teleworking tends to be more conditioned by structural factors of employment, such as sector of activity, educational level and type of contract, than by personal or family situation. In other words, the possibility of teleworking depends more on the characteristics of the job than on the fact of living alone or with other people.<br><br>Secondly, the flexibility of teleworking allows both singles and people in couples or with dependents to adapt to this modality without their cohabitation structure representing a significant barrier. Unlike other work dynamics that may be more influenced by household composition, telework appears to be similarly accessible to different types of households.<br>Likewise, these results could reflect a greater diversification of profiles within teleworking, in which different cohabitation groups manage to insert themselves without their personal situation clearly determining their participation in this type of work [line 257 and following]<br><br>This suggests that access to telework is not evenly distributed across all educational levels but is conditioned by educational background. It is possible that occupations that allow or facilitate this type of work require specialized skills, higher qualifications or belong to sectors where the use of digital technologies is essential. In this sense, telework could be reinforcing pre-existing inequalities in the labor market, favoring those with higher education and limiting access for those with lower educational levels [line 260 and following]<br><br>These results suggest that telework is not only linked to occupation, but also to job stability and workload. People with temporary contracts and reduced working hours, who tend to be in more precarious positions, have less access to this modality, which reinforces pre-existing labor inequalities. In addition, the fact that women have a greater presence in teleworking within these groups could be related to patterns of work-life balance and occupational segmentation, which raises questions about how teleworking may be reproducing gender dynamics in the labor market [line 271 and following]
10. Gender Gaps in Teleworking<br>Across the entire sample, the gender gap in teleworking adoption is relatively small (2.2 percentage points in favor of women). However, among full-time workers, the gender gap increases to 5 percentage points. Additionally, the probability of part-time work is 15 percentage points higher for women. In households with children under 5, 42% of women work part-time compared to only 9% of men. In these households, gender differences in teleworking probabilities are minimal. However, as children age, the likelihood of part-time work decreases slightly among women (35% in households with children aged 6–11 and 26% in those with children aged 12–15). At these ages, the gender gap in teleworking rises to 6–7 percentage points. These findings raise doubts about the authors' conclusion that work-life balance is not a significant factor in teleworking adoption.	Work-life balance is indeed an important factor. However, it is an explanatory factor with less weight than others found in this research. Nevertheless, this observation is accepted and is included in future lines of work according to the following text:<br><br>Another valuable line of research would be to detail how these gender differences in telework relate to family composition, particularly in households with children. In this sense, it could be of particular interest to study the impact of telework as a function of the age of the children, since care needs and family organization vary significantly according to this variable. This approach would make it possible to identify more precisely how teleworking influences family and work responsibilities, and how these interactions may be a key factor in perpetuating or reducing gender inequalities in the work and domestic spheres. However, these questions are beyond the scope of this paper and are proposed as future lines of research [line 496 and following].
11. Potential Additional Analyses<br>A possible avenue for exploration could involve focusing on the sample of full-time workers or combining the effects of part-time work (predominantly female) in the unequal development of teleworking. Descriptive tables are attached.	The observation is accepted and included as a line of work in future research according to the following text:<br><br>This research opens interesting avenues of work that allow further deepening the understanding of telework as a social and labor phenomenon. In particular, it would be relevant to explore in more detail the unequal development of teleworking from a gender perspective, considering the differences that may arise depending on the type of work dedication, whether full or part-time. Future research could delve deeper into how work dynamics in telework environments affect men and women differently, revealing possible structural inequalities and specific patterns that have not been addressed in depth in this paper. [line 496 and following].
Policy Recommendations<br>The manuscript claims that teleworking has positive effects,	It should be noted that the observations related to stress and flexibility do not represent an empirical finding derived from this work but are part of the theoretical framework that underpins the analysis of the causes and

such as reduced stress and increased flexibility. Where are the tables investigating this? Similarly, policy recommendations are made on issues not analyzed in the article.	consequences of telework. These considerations are supported by previous studies that have identified how telework dynamics can influence both the perception of control and autonomy, and the emergence of stresses associated with work-life balance, providing a theoretical context for interpreting the results obtained in this research.<br>Recommendations that were not part of the analysis have been eliminated.<br>New text: In this regard, it is essential to promote actions that create new opportunities for all individuals, with a particular focus on achieving gender equality in the workplace. This involves not only removing obstacles that hinder women's participation in economic activities but also implementing social protection measures and closing gaps in professional environments, ensuring fair and equitable access to telework opportunities.
13. Clarity and Presentation<br>The wording of some phrases is ambiguous:<br>a. Occupation appears as a factor influencing teleworking propensity (Asmussen et al., 2024). Why? Can the mechanisms making occupation relevant to teleworking be explained?	Text A has been replaced by Text B (see line 82):<br>Text A:<br>In any case, occupation appears to be a factor to be considered in the propensity to telework (Asmussen et al., 2024).<br>Text B:<br>In any case, occupation and job characteristics seem to be a factor to be considered in the propensity to telework, as factors such as the digitization of tasks, the use of specialized tools, the need for face-to-face interaction and security requirements influence its viability. Knowledge-based occupations, such as programming or accounting, can easily adapt to remote work, while those requiring physical presence, such as manufacturing or face-to-face healthcare, present greater limitations. In addition, autonomy in work management and the need for supervision also play a key role in the effectiveness of telecommuting. Several studies have analyzed what proportion of jobs can be performed remotely, concluding that differences in the feasibility of telework depend primarily on the characteristics of each occupation (Asmussen et al., 2024; Dingel and Neiman, 2020).<br><br>Besides, next reference has been added:<br>Dingel, J. I. and Neiman, B. (2020). How many jobs can be done at home? Journal of Public Economics,189, 104235. https://doi.org/10.1016/j.jpubeco.2020.104235
13. Clarity and Presentation<br>The wording of some phrases is ambiguous:<br>b. Similarly, education is noted as a factor to consider (Kley and Reimer, 2023). The reasons for its importance should be clarified, outlining how education affects teleworking.	Text A has been replaced by Text B (see line 92):<br>Text A:<br>Linked to this issue, education is a variable that must be taken into account. (Kley and Reimer, 2023).<br>Text B:<br>Related to this issue, education is a key factor in telework because it influences adaptation to new technologies, autonomy in time management and the ability to solve problems independently. According to Kley and Reimer (2023), workers with higher levels of education tend to perform better in telework environments because they possess advanced digital skills and greater organizational capacity, allowing them to maintain high levels of productivity without direct supervision. In addition, education fosters cognitive flexibility, facilitating the transition to telecommuting-based work models (Chowhan, 2021).
13. Clarity and Presentation<br>Additionally, the presentation could be improved by numbering manuscript sections, completing tables with standard logistic regression statistics and sample sizes, and including titles and sources for the two final graphs.	The presentation has been improved by numbering manuscript sections, completing tables with standard logistic regression statistics and sample sizes, and including titles and sources for the two final graphs.<br><br>The following amendment has been introduced in Table 1 (see line 164):<br>Note: The values correspond to the subtraction of the percentage of men and women who telework. A positive value indicates that the proportion of men is higher than women one, while a negative value indicates that the proportion of women is higher than men one.

Reviewer #2

Suggestion	Comment
Despite the usefulness of the paper, it should undertake several diagnostic tests to examine basic logistic regression assumptions. In logistic regression, basic assumptions must be met, such as the independence of errors, the absence of multicollinearity and the lack of outliers. This can be easily achieved using any statistical or econometric software to enhance the quality of the paper. At present, the paper assumes all these assumptions are fulfilled. Although the findings can still be used where assumptions were violated, this could be explained to the readership.	Firstly, multicollinearity has been verified using the GVIF coefficient, whose values are reported in the tables of the study, confirming the absence of serious problems of collinearity between the explanatory variables.<br><br>Secondly, a logistic regression residual analysis was conducted to verify the validity of the logistic regression and the fulfillment of its assumptions. We have examined the behavior of the residuals using diagnostic plots, which show that the model is correctly specified and there are no systematic patterns suggesting fit problems.<br>The residual plots included in the analysis confirm that there are no indications of serious violations of the independence assumptions, absence of collinearity or poor model fit. In addition, the absence of influential outliers that could distort the estimates has been verified.<br><br>Thus, Graph 3 was included along with next text (see line 559):<br>Finally, an evaluation of the residuals of the logit model is conducted (Graph 3). It is observed that the residuals behave in accordance with the expected values, with no deviations identified, absence of collinearity or poor model fit.<br><br>Finally, in "Material and methods" section was included the next paragraph (see line 244):<br>Finally, an evaluation of the residuals of the logit model is conducted to verify the validity of the logistic regression and to determine whether the assumptions underlying the regression are fulfilled.
The other similar limitation of the paper is the assumption of linearity between the dependent variable and independent variables. This should be tested empirically and completed as a minor revision using diagnostic statistics to prove the linear relationship. Moreover, when the relationship is not linear, the regression findings should also be amended.	The assumption of linearity between the response variable and the logit of the independent variables has been verified through residual analysis. Specifically, we examined diagnostic plots of the residuals to ensure that no systematic patterns were present, indicating that the model specification is appropriate.
The scale of the measurement should also be discussed more rigorously and clearly, such as which variables are on continuous scales and which ones are dichotomous, nominal, ordinal, interval or ratio. At the moment, the information for the readership on the data is limited.	The measurement scales of different variables was pointed out (see lines 199, 208, 209, 216, 218, 224, 228 and 229)
Are regression estimations weighted ones? If not, it is advisable to use sample weights (inflation factors) to make the results representative. Does the survey provide sample weights?	The weights provided by the EWCTS survey are designed to ensure representativeness of the European population, correcting for possible biases derived from the sample design and non-response. However, given that our analysis focuses exclusively on Spain, the application of these weights may not be appropriate. The sampling structure and nonresponse patterns may differ within the Spanish subgroup, which would make the weights designed at the European level not correctly reflect the distribution of the Spanish population.<br>Furthermore, the use of these weights in a smaller sample such as ours carries a significant risk of inflating the type I error rate (false positives) by artificially reducing variances and making virtually all variables appear statistically significant. This is because the weights amplify the contribution of certain observations, affecting the estimation of standard errors in regression models.<br>According to methodological references, in studies whose main objective is to analyze relationships between variables, rather than to produce descriptive population estimates, the inclusion of weights is not always necessary and can even be detrimental if the weights have not been designed specifically for the subgroup analyzed. A valid alternative is to correctly specify the model by including the variables that affect the probability of response, which we have done by incorporating factors such as age, sex, region, occupation and sector in the analysis, as recommended by the EWCTS itself.
All research has limitations. Prior to the 'Conclusion', please ensure to have a limitations section.	Accepted and a text on limitations and future lines of research has been included.
The conclusion section should be written separately	The discussion and conclusions sections have been separated into
