# Supplementary material for: Importance of occupation in the increase of gender differences in the advance of telework in Spain
Source: PLoS One. 2025 May 7;20(5):e0322847. doi: 10.1371/journal.pone.0322847 (PMC12058165; doi:10.1371/journal.pone.0322847)
Supplement: Attachment — Submitted filename: Response_to_Reviewers_auresp_2.pdf [file pone.0322847.s003.pdf]

Responses to editor & reviewers

Suggestion	Comment
Expand the introduction to provide an enhanced analysis of the study's purpose and motives that drive the research question and hypotheses	Taking into account the particularity of the Spanish case and the existing literature on telework from a gender perspective, this research adopts a cross-sectional approach. Using data from the European Working Conditions Survey, we seek to validate hypotheses on the moderating effect of the propensity to telework as a function of variables such as education, life stage (family structure and cohabitation) and work history (occupation, type of contract and working hours).<br>This study provides a novel perspective by analyzing how these factors influence the adoption of telework within the specific structure of the Spanish labor market. In doing so, it fills a gap in the literature by addressing insufficiently explored dimensions. In addition, its findings may contribute to the development of new theories on telework, as well as to the identification of areas that require further analysis. Finally, the results of this research can serve as a basis for the design of more effective policies that promote better working conditions, thereby fostering improved social welfare.<br><br>[line 215 and following]
- Improve readability by simplifying complex sentences.<br>- Extensive proofreading must be carried out to address all grammar inconsistencies, including the mixed usage of American and British English.	In accordance with the instructions provided, the manuscript has undergone a comprehensive revision to rectify all grammatical inconsistencies, including the utilisation of both American and British English.<br>It is asserted that this has resulted in a significant enhancement of the readability of the text.
Add a brief explanation regarding how data limitations affect the results and how they were addressed	This reduction was partly due to the limitations inherent in the EWCS dataset, such as the multi-level structure of responses, which in some cases did not align directly with the variables in the model. In order to address this issue and prevent systematic errors or biases resulting from ambiguous response assignment, cases that could not be accurately categorized were excluded. This approach was adopted to ensure the validity of the results obtained by avoiding the artificial inflation of the sample size.<br><br>In order to address the issue of missing data, a structured approach was adopted. Initially, an assessment was conducted to determine the

	extent and pattern of missing values in key variables. Cases with missing values in the dependent variable (teleworking status) were excluded, as they could not contribute to the analysis. For the independent variables, a complete-case analysis (listwise deletion) was applied, with observations retained only if complete data was available for all predictors. The selection of this method was driven by the necessity to preserve both the consistency and interpretability of the model, whilst ensuring that the final sample accurately reflected the population under study.<br><br>A sensitivity check was conducted to compare key characteristics (such as gender, occupation, and education level) between the full Spanish sample (2,903) and the final analytical sample (1,320). The distributions remained largely consistent, suggesting that the exclusion of incomplete cases did not introduce substantial bias.<br><br>[line 240 and following]
- Enhance policy discussion to highlight implications for labor regulations.<br>- A paragraph dealing with policy recommendations must be added	The results of this research can serve as a basis for the formulation of more equitable labor policies adapted to the needs of workers at different stages of their professional and personal lives. Although teleworking is regulated in detail in Spain as of Law 10/2021, of July 9, on telecommuting, studies such as this one can provide evidence for the revision of regulations on labor flexibility, work-life balance, and the protection of workers' rights in teleworking contexts. It is necessary to bear in mind that regulations must adapt to the changing reality of employment formulas, so research such as this can help to inform regulatory adaptations on the subject. They can also guide the design of measures to reduce gender inequalities in the access and use of telework, ensuring that this does not deepen pre-existing gaps in the labor market.<br><br>[line 674 and following]<br><br>Reference related to Ley 10/2021 has been included in References section:<br>Ley 10/2021, de 9 de julio, de trabajo a distancia, BOE, 164, Referencia: BOE-A-2021-11472<br><br>[line 873]
